# Risk prediction of diabetic retinopathy based on visit-to-visit fasting blood glucose indices

**DOI:** 10.3389/fendo.2024.1420948

**Published:** 2024-09-20

**Authors:** Ying Ju, Zhengyang Guo, Jiaqi Ai, Kai Yang, Xiaoxuan Zhu, Keai Shi, Chunmei Li, Tianyun Yu, Yunfan Xiao, Binbin Su, Jinxia Yan, Ziyu Li, Wei Lian, Zhenqin Wang, Shasha Ding, Yudie Wang, Fan Lu, Lele Cui, Ming Li

**Affiliations:** ^1^ National Clinical Research Center for Ocular Diseases, Eye Hospital, Wenzhou Medical University, Wenzhou, China; ^2^ School of Optometry and Vision Science, University of New South Wales, Sydney, NSW, Australia

**Keywords:** FBG index, risk prediction, diabetic retinopathy, cohort study, diabetic microvascular complication

## Abstract

**Objective:**

The long-term glucose monitoring is essential to the risk assessment of diabetic retinopathy (DR), the aim of this study was to investigate the predictive ability of visit-to-visit fasting blood glucose (FBG) indices on the risk of DR.

**Methods:**

This was a community-based, cohort study conducted from 2013 to 2021. DR was diagnosed by digital fundus photography. The FPG indices included FBG, var. Associations of each FBG indices and DR were estimated using multinomial logistic regression models adjusting for confounders, and discrimination was determined by area under the curve (AUC). Predictive utility of different models was compared by changes in AUC, integrated discrimination improvement (IDI), and net reclassification index (NRI).

**Results:**

This study analyzed 5054 participants, the mean age was 46.26 ± 11.44 years, and 2620 (51.84%) were women. After adjustment for confounders, the adjusted odds ratios (ORs) with 95% confidence intervals (CIs) for FBG, SD, CV, VIM, ARV, M-FBG, and cumulative FBG load were 1.62 (1.52—1.73), 2.74 (2.38—3.16), 1.78 (1.62—1.95), 1.11 (0.95—1.29), 1.72 (1.56—1.91), 2.15 (1.96—2.36), and 2.57 (2.31—2.85), respectively. The AUC of the model with separate cumulative FBG load and classical risk factors was 0.9135 (95%CI 0.8890—0.9380), and no substantive improvement in discrimination was achieved with the addition of other FBG indices once cumulative FBG load was in the model.

**Conclusions:**

Cumulative FBG load is adequate for capturing the glucose-related DR risk, and the predictive utility of cumulative FBG load is not significantly improved by adding or replacing other FBG indices in the assessment of DR risk.

## 1 Introduction

As the most common and specific microvascular complication of diabetes, diabetic retinopathy (DR) remains a leading cause of preventable vision impairment and blindness in working-age adults (1–4). The global diabetes prevalence in adults aged 20—79 years is expected to rise to 12.2% (783.2 million) by 2045, as estimated (5). Accordingly, the annual incidence of DR ranged from 2.2% to 12.7% and progression from 3.4% to 12.3%, respectively (6). Therefore, early identification of the onset of DR and further active and effective interventions to delay progression are essential to reduce DR-related risks.

Previous studies have established that long-term, sustained hyperglycemia is a key risk factor for DR (7). Furthermore, strong evidence suggests that intensive glucose control achieved through medication or therapy effectively prevents DR onset or delays its progression (8–10). Fasting blood glucose (FBG), a common metric for monitoring glycemic control, captures immediate blood glucose levels. Studies on the relationship between FBG levels and DR have primarily relied on single FBG data. Due to fluctuations, FBG monitoring at a single point may not capture long-term trends, reducing accuracy of DR risk assessment. Therefore, tracking FBG levels over time can provide a more reliable assessment of DR risk.

Recently, several visit-to-visit FBG indices, such as standard deviation (SD), coefficient of variation (CV), variation independent of the mean (VIM), average real variability (ARV), mean fasting blood glucose level (M-FBG), and cumulative FBG load, were calculated from multiple readings of FBG and documented to be associated with diabetic (macrovascular and microvascular) complications (11–15). However, most previous studies have focused on the relationship between FBG indices and cardiovascular complications (16, 17), diabetic nephropathy (18), and diabetes peripheral neuropathy (13, 14), and there are only few studies on DR (19, 20). Therefore, there is a need to explore whether these FBG indices can be used as predictors of DR risk and further identify the most informative predictors of these FBG indices in terms of DR risk.

Therefore, our study aimed to investigate the separate and joint predictive ability of different FBG indices for the risk of DR, thereby identifying DR and providing a robust basis for further glycemic control.

## 3 Methods

### 3.1 Study population

The data used in this study were obtained from the Jidong Eye Cohort Study (JECS). The JECS design was recorded as previously described. The participants were the general population consecutively recruited from the Jidong community (Tangshan City, northern China) from July 2013 to August 2014. From 2013 to 2021, the participants underwent five health screenings every one or two years. Routine screening included comprehensive laboratory tests (blood biochemistry and routine blood examinations) and a standardized questionnaire interview regarding demographic characteristics and medical history. Following routine screening, all participants underwent a comprehensive ophthalmological examination. Participants with less than three FBG tests, those lacking FBG tests from May 2019 to November 2021, and those with missing or unqualified fundus photography were excluded from the analysis (21, 22). This resulted in a final sample size of 5054 subjects for final analysis, as shown in online **Supplementary Figure 1**.

This study complied with the principles of the Declaration of Helsinki (revised in 2013). It was approved by the Ethics Committee of the Staff Hospital of Jidong Oil-field of Chinese National Petroleum (approval document 2018 YILUNZI 1) and the Ethics Committee of Wenzhou Medical University Affiliated Ophthalmology Hospital (2021-074-K-63-01). All subjects signed the informed consent.

### 3.2 Clinical and biological parameters

In this study, age, sex, educational level, income, smoking and drinking status, history of comorbidities, and current medication use were recorded using a standardized questionnaire. All participants underwent a comprehensive physical examination and laboratory tests. The education level was categorized into: “illiteracy or primary school or middle school” and “college graduate or above”. The average monthly income was categorized into “≤ ¥5,000” and “> ¥5,000”. In this study, hypertension was defined as systolic blood pressure (SBP) ≥ 140 mmHg, or diastolic blood pressure (DBP) ≥ 90 mmHg, or self-reported hypertension history, or current use of antihypertensive medications. Dyslipidemia was defined by either low-density lipoprotein (LDL-C) ≥ 3.37 mmol/L, high density lipoprotein (HDL-C) < 1.04 mmol/L, total cholesterol (TC) ≥ 5.18 mmol/L, triglyceride (TG) ≥ 1.7 mmol/L, self-reported history of dyslipidemia, or current use of lipid-lowering medications.

### 3.3 FBG collection and calculation of longitudinal FBG indices

Fasting plasma glucose levels were measured in the early morning after at least 8 hours of food and water deprivation. Blood samples were collected from the antecubital vein (elbow vein). Following storage, the fasting plasma glucose levels were measured using an autoanalyzer employing the glucose oxidase method. The following four indices representing long-term glycemic variability were calculated: 1) SD: the standard deviation of FBG values; 2) CV: CV (%) = SD (mmol/L)/mean (mmol/L) ×100% 3) VIM: VIM= 100×SD/mean^β^, β is the regression coefficient based on the ln of the SD over the ln of the mean; 4) ARV (23, 24). In our study, M-FBG was calculated as the average of the FBG values measured over time. Additionally, cumulative FBG load was determined by dividing the area under the curve (AUC) for FBG values ≥ 5.6 mmol/L divided by the AUC for all FBG values and then multiplied by 100 to achieve the percentage (25, 26).

### 3.4 Ophthalmic examination

All participants in our study underwent a complete ophthalmological examination between May 2019 and November 2021, including best-corrected visual acuity (BCVA) using a standard logarithmic visual acuity chart, the status of refraction using an auto refractometer (KR800; Topcon; Tokyo, Japan), axial length (AL) using a Lenstar 900 (Haag-Streit; Koeniz, Switzerland), and optical coherence tomography angiography (OCTA) images using a spectral-domain OCTA (RTVue XR Avanti with AngioVue; Optovue; Fremont, CA, United States). At least two independent ophthalmologists reviewed all the examination results. Digital fundus photography of each eye was performed by a trained ophthalmologist using a 45°non-mydriatic fundus camera (CR2AF; Canon; Tokyo, Japan). For image quality control, two trained ophthalmologists ensured that the images qualified for further analysis. Qualified fundus photographs were read by two experienced ophthalmologists double-blind, according to the International Clinical Diabetic Retinopathy (ICDR) Severity Scale (27). The diagnosis of DR was confirmed using digital fundus photography.

### 3.5 Statistical analysis

Continuous variables are expressed as mean (SD), as they were almost normally distributed, and categorical variables were expressed as numbers and percentages. Differences in baseline characteristics between the groups were compared using unpaired t-test or Wilcoxon rank sum test for continuous variables, and chi-square test or Fisher’s exact test for categorical variables. Missing data were handled differently depending on the variable. For continuous variables like body mass index (BMI), we replaced missing values with the mean. For categorical variables like current smoking, current drinking, and hypertension, we used the median as the replacement value. The proportions of missing data for all covariates before imputation were less than 10%. Associations between different FBG indices were assessed using Spearman’s correlations, both unadjusted and then sex and age were considered. Multinomial logistic regression models were used to estimate the relationship between each FBG index and DR. The DR models were adjusted for age, sex, educational level, income, current smoking, current drinking, hypertension, and dyslipidemia. The AUCs were used to assess the discrimination of different models with FBG indices. Changes in the AUC, integrated discrimination improvement (IDI) and net reclassification index (NRI) were calculated to compare the predictive ability of different models for the risk of DR. In addition, changes in Akaike Information Criteria (AIC) and Bayesian Information Criteria (BIC) were used to assess the improvement in goodness of model fit. We performed sensitivity analyses in subjects with more than three FBG tests and more than four FBG tests.

We expressed associations by βs and 95% confidence intervals (CIs) for all analyses. 2-tailed P values < 0.05 were considered statistically significant. All statistical analyses were performed using SAS software (version 9.4; SAS Institute Inc., Cary, NC, USA) and R 4.3.2(Packages included).

## 4 Results

### 4.1 Baseline characteristics

A total of 5054 participants with a mean age of 46.26 years (SD 11.44) were included in the final analysis, of whom 2620 (51.84%) were women. **Table 1** shows the baseline characteristics of NO DR and DR groups. DR was observed in 158 (3.13%) participants. Participants in the DR group were more male, older and less educated, more likely to be current smokers and drinkers, had a higher prevalence of hypertension and hyperlipidemia, and had higher levels of BMI, FBG, SD, CV, ARV, M-FBG, and cumulative FBG load (**Table 1**).

**Table 1:** Participant characteristics at baseline.

Characteristics	Total (n=5054)	No DR (n=4896)	DR (n=158)	*P* value
Age, years	46.26(11.44)	46.00(11.38)	54.16(10.49)	<0.001
Female, n(%)	2620 (51.84)	2557 (52.23)	63 (39.87)	0.002
Educational level, n(%)				<0.001
Illiteracy/Primary School/Middle School	1472 (29.13)	1389 (28.37)	83 (52.53)	
College/University	3582 (70.87)	3507 (71.63)	75 (47.47)	
Income, n(%)				0.04
≤5000	4096 (81.04)	3958 (80.84)	138 (87.34)	
>5000	958 (18.96)	938 (19.16)	20 (12.66)	
Current smoking, n(%)	889 (17.59)	843 (17.22)	46 (29.11)	<0.001
Current drinking, n(%)	1096 (21.69)	1046 (21.36)	50 (31.65)	0.002
Hypertension, n(%)	1339 (26.49)	1249 (25.51)	90 (56.96)	<0.001
Dyslipidemia, n(%)	2923 (57.84)	2795 (57.09)	128 (81.01)	<0.001
BMI, kg/m²	24.57(3.46)	24.52(3.46)	25.93(3.40)	<0.001
FBG, mmol/L	5.83(1.48)	5.72(1.20)	9.31(3.59)	<0.001
SD, mmol/L	0.63(0.59)	0.59(0.48)	1.87(1.57)	<0.001
CV, %	10.51(6.18)	10.21(5.59)	19.97(12.83)	<0.001
VIM, %	0.76(0.32)	0.76(0.31)	0.79(0.40)	0.18
ARV, %	13.36(7.71)	13.06(7.13)	22.71(15.48)	<0.001
M-FBG, mmol/L	5.66(1.09)	5.56(0.87)	8.50(2.57)	<0.001
cumulative FBG load, %	5.76(9.73)	4.95(8.10)	30.74(18.70)	<0.001

Data are presented as n (%) or means ± SD.

DR, diabetic retinopathy; BMI, body mass index; FBG, fasting blood glucose; SD, standard deviation; CV, coefficient of variation; VIM, variation independent of the mean; ARV, average real variability; M-FBG, mean fasting blood glucose level.

Participants with higher FBG, SD, CV, ARV, M-FBG, and cumulative FBG load levels were more likely to be men, less educated, current smokers, current drinkers, and had a higher prevalence of hypertension and dyslipidemia (**Supplementary Tables 1**-**7**). The M-FBG and cumulative FBG load were highly correlated with FBG (r > 0.6) (**Supplementary Table 8**).

### 4.2 Multivariable association of different FBG indices with DR outcomes

**Table 2** shows the relationships between different FBG indices and DR. The adjusted odds ratios (ORs) with 95%CIs for FBG, SD, per 1 SD increase in CV, per 1 SD increase in VIM, per 1 SD increase in ARV, M-FBG, and per 1 SD increase in cumulative FBG load were 1.62(1.52—1.73), 2.74(2.38—3.16), 1.78(1.62—1.95), 1.11(0.95—1.29), 1.72(1.56—1.91), 2.15(1.96—2.36), and 2.57(2.31—2.85), respectively, after adjusting for age, sex, educational level, income, current smoking, current drinking, hypertension and dyslipidemia. Specifically, the SD and per 1 SD increase in the cumulative FBG load showed stronger links to DR.

**Table 2:** Associations of different FBG indices with DR in the logistic regression model.

	OR (95%CI)	Adjusted OR (95%CI)
FBG	1.73(1.63, 1.85)	1.62(1.52, 1.73)
SD	3.17(2.74, 3.66)	2.74(2.38, 3.16)
CV, per 1 SD Increase	1.93(1.75, 2.11)	1.78(1.62, 1.95)
VIM, per 1 SD Increase	1.11(0.95, 1.29)	1.11(0.95, 1.29)
ARV, per 1 SD Increase	1.84(1.66, 2.03)	1.72(1.56, 1.91)
M-FBG	2.35(2.15, 2.57)	2.15(1.96, 2.36)
cumulative FBG load, per 1 SD Increase	2.78(2.52, 3.07)	2.57(2.31, 2.85)

FBG, fasting blood glucose; DR, diabetic retinopathy; OR, odds ratio; CI, confidence interval; SD, standard deviation; CV, coefficient of variation; VIM, variation independent of the mean; ARV, average real variability; M-FBG, mean fasting blood glucose level.

Adjusted for age, sex, educational level, income, current smoking, current drinking, hypertension, dyslipidemia, body mass index.

### 4.3 Prediction of DR in addition to classical risk factors

**Figure 1:**
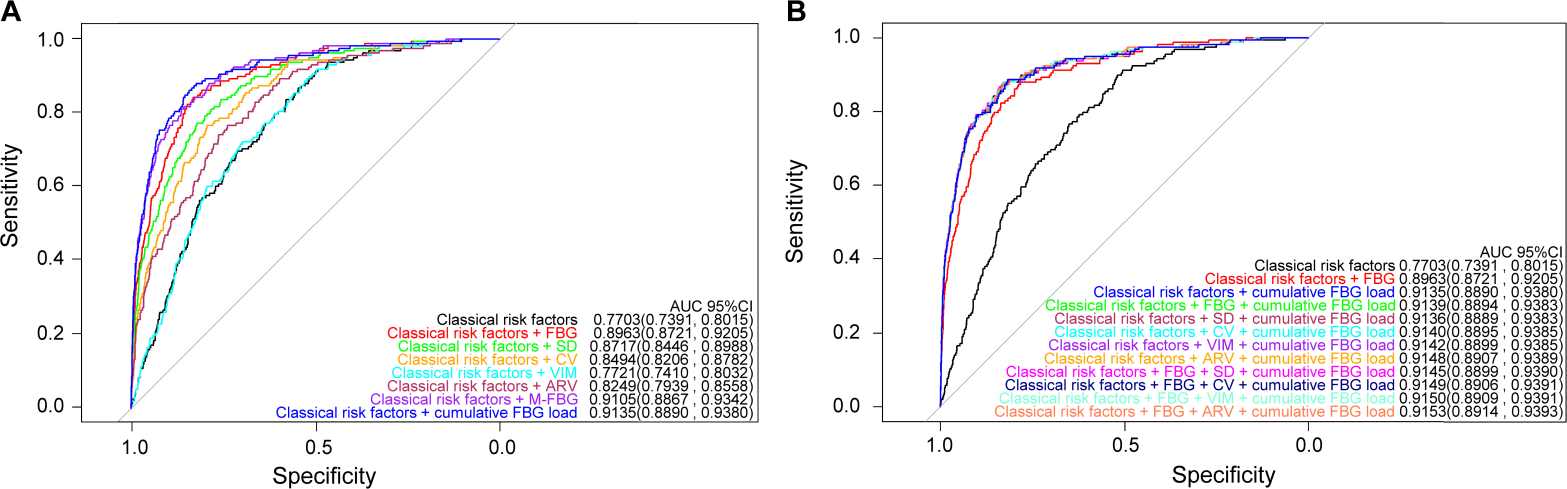
Prediction performance of the models. **(A)** Models with a separate FBG index. **(B)** Models with combined FBG indices.

Classical risk factors alone achieved reasonable discrimination for DR prediction (AUC 0.7703, 95%CI 0.7391—0.8015; **Figure 1A**). Adding any FBG index, except the VIM, further improved discrimination (**Figure 1A**). Among models with individual FBG index, discrimination and reclassification increased only when M-FBG or cumulative FBG load was added compared to the model with FBG (**Tables 3**; **4**), and the model with separate cumulative FBG load achieved the highest discrimination (AUC 0.9135, 95%CI 0.8890-0.9380; **Figure 1A**, **Table 3**). When adding ARV or FBG and ARV to the model with separate cumulative FBG load, the discrimination improved modestly (changes in AUC +0.0013, 95%CI 0.0001—0.0025 and +0.0018, 95%CI 0.0004—0.0033; **Figure 1B**, **Supplementary Table 12**). However, adding ARV or FBG and ARV did not further improve the reclassification (**Supplementary Table 13**). There was no compelling evidence that adding other indices after adding the cumulative FBG load improved the goodness of model fit as measured by AIC and BIC (**Supplementary Table 14**).

**Table 3:** Discrimination statistics for prediction of DR compared with the model with classical risk factors and FBG (n=5054).

Models	AUC (95%CI)	Changes in AUC(95%CI)	P value
Classical risk factors + FBG	0.8963(0.8721, 0.9205)	Reference	
Classical risk factors +SD	0.8717(0.8446, 0.8988)	-0.0246(-0.0403, -0.0089)	0.002
Classical risk factors +CV	0.8494(0.8206, 0.8782)	-0.0469(-0.0667, -0.0272)	<0.001
Classical risk factors +VIM	0.7721(0.7410, 0.8032)	-0.1242(-0.1504, -0.0980)	<0.001
Classical risk factors +ARV	0.8249(0.7939, 0.8558)	-0.0714(-0.0940, -0.0489)	<0.001
Classical risk factors +M-FBG	0.9105(0.8867, 0.9342)	0.0142(0.0043, 0.0240)	0.005
Classical risk factors +cumulative FBG load	0.9135(0.8890, 0.9380)	0.0172(0.0042, 0.0302)	0.009
Classical risk factors + FBG + SD	0.8993(0.8754, 0.9231)	0.0030(-0.0022, 0.0082)	0.26
Classical risk factors + FBG + CV	0.8958(0.8714, 0.9201)	-0.0005(-0.0012, 0.0002)	0.13
Classical risk factors + FBG + cumulative FBG load	0.9139(0.8894, 0.9383)	0.0176(0.0049, 0.0302)	0.007
Classical risk factors + SD + cumulative FBG load	0.9136(0.8889, 0.9383)	0.0173(0.0039, 0.0307)	0.01
Classical risk factors + CV + cumulative FBG load	0.9140(0.8895, 0.9385)	0.0177(0.0046, 0.0308)	0.008
Classical risk factors + ARV + cumulative FBG load	0.9148(0.8907, 0.9389)	0.0185(0.0056, 0.0315)	0.005
Classical risk factors + FBG + SD + cumulative FBG load	0.9145(0.8899, 0.9390)	0.0182(0.0052, 0.0311)	0.006
Classical risk factors + FBG + CV + cumulative FBG load	0.9149(0.8906, 0.9391)	0.0186(0.0059, 0.0312)	0.004
Classical risk factors + FBG + VIM + cumulative FBG load	0.9150(0.8909, 0.9391)	0.0187(0.0062, 0.0312)	0.003
Classical risk factors + FBG + ARV + cumulative FBG load	0.9153(0.8914, 0.9393)	0.0190(0.0065, 0.0316)	0.003

Classical risk factors: age, sex, BMI, educational level, income, current smoking, current drinking, hypertension, dyslipidemia.

DR, diabetic retinopathy; FBG, fasting blood glucose; AUC, area under the curve; CI, confidence interval; SD, standard deviation; CV, coefficient of variation; VIM, variation independent of the mean; ARV, average real variability; M-FBG, mean fasting blood glucose level.

**Table 4:** Reclassification statistics for prediction of DR compared with the model with classical risk factors and FBG (n=5054).

Models	IDI (95% CI)	P value	NRI(Categorical) (95%CI)	P value
Classical risk factors + FBG	Reference		Reference	
Classical risk factors +SD	-0.0372(-0.0651, -0.0093)	0.009	-0.0751(-0.1465, -0.0037)	0.04
Classical risk factors +CV	-0.0861(-0.1173, -0.0549)	<0.001	-0.1615(-0.2292, -0.0938)	<0.001
Classical risk factors +VIM	-0.1618(-0.2016, -0.1221)	<0.001	-0.2442(-0.3120, -0.1763)	<0.001
Classical risk factors +ARV	-0.1030(-0.1413, -0.0647)	<0.001	-0.1788(-0.2530, -0.1047)	<0.001
Classical risk factors +M-FBG	0.0501(0.0215, 0.0787)	<0.001	0.1190(0.0418, 0.1963)	0.003
Classical risk factors +cumulative FBG load	0.0603(0.0312, 0.0894)	<0.001	0.1162(0.0295, 0.2028)	0.009
Classical risk factors + FBG + SD	-0.0003(-0.0053, 0.0048)	0.92	0.0000(-0.0248, 0.0248)	1.00
Classical risk factors + FBG + CV	0.0005(0.0000, 0.0010)	0.05	0.0000(0.0000, 0.0000)	1.00
Classical risk factors + FBG + cumulative FBG load	0.0614(0.0347, 0.0881)	<0.001	0.1288(0.0482, 0.2095)	0.002
Classical risk factors + SD + cumulative FBG load	0.0614(0.0299, 0.0928)	<0.001	0.1411(0.0502, 0.2320)	0.002
Classical risk factors + CV + cumulative FBG load	0.0623(0.0315, 0.0931)	<0.001	0.1407(0.0515, 0.2299)	0.002
Classical risk factors + ARV + cumulative FBG load	0.0618(0.0321, 0.0915)	<0.001	0.1280(0.0383, 0.2177)	0.005
Classical risk factors + FBG + SD + cumulative FBG load	0.0645(0.0368, 0.0922)	<0.001	0.1543(0.0674, 0.2413)	<0.001
Classical risk factors + FBG + CV + cumulative FBG load	0.0652(0.0376, 0.0928)	<0.001	0.1664(0.0800, 0.2527)	<0.001
Classical risk factors + FBG + VIM + cumulative FBG load	0.0643(0.0370, 0.0916)	<0.001	0.1415(0.0576, 0.2254)	<0.001
Classical risk factors + FBG + ARV + cumulative FBG load	0.0630(0.0358, 0.0901)	<0.001	0.1278(0.0434, 0.2122)	<0.001

Classical risk factors: age, sex, BMI, educational level, income, current smoking, current drinking, hypertension, dyslipidemia.

DR, diabetic retinopathy; FBG, fasting blood glucose; IDI, integrated discrimination improvement; CI, confidence interval; NRI, net reclassification improvement indexes; SD, standard deviation; CV, coefficient of variation; VIM, variation independent of the mean; ARV, average real variability; M-FBG, mean fasting blood glucose level.

Specifically, among all the models, the highest discrimination was observed when the FBG, ARV, and cumulative FBG load were added (AUC 0.9153, 95%CI 0.8914—0.9393]; **Supplementary Table 9**). Compared to the model with separate FBG, the discriminatory power and risk reclassification of model with FBG, ARV, and cumulative FBG load improved significantly (IDI 0.0630, 95%CI 0.0358—0.0901; NRI 0.1278, 95%CI 0.0434—0.2122; **Supplementary Table 10**). The goodness of model fit improved significantly as well (ΔAIC -99.6533; ΔBIC -86.5970; **Supplementary Table 11**). However, when compared to the model with separate cumulative FBG load, the discriminatory power and risk reclassification did not improve substantially (IDI 0.0027, 95%CI -0.0022—0.0076; NRI -0.0006, 95%CI -0.0399—0.0386; **Supplementary Table 13**), and the goodness of model fit showed the opposite trend (ΔAIC 1.9209; ΔBIC 14.9770; **Supplementary Table 14**).

### 4.4 Sensitivity analysis

**Supplementary Tables 15**, **16** show the discrimination of the different models among participants (n=3557 individuals) with more than three FBG tests and participants (n=1587 individuals) with more than four FBG tests. The AUCs with 95%CIs of the models with separate cumulative FBG load were 0.9209, 0.8937—0.9480 and 0.9317, 0.9004—0.9631. We observed similar discrimination in the three groups.

## 5 Discussion

In this study, we evaluated the predictive ability of various FBG indices for DR. This study showed that SD and per 1 SD increase in cumulative FBG load had stronger associations with the risk of DR among these FBG indices. In addition, compared with other FBG indices, cumulative FBG load was a better predictor of DR. AUC analysis clearly showed that the model with separate cumulative FBG load was sufficiently qualified to capture the glucose-related DR risk. The predictive ability of model with separate cumulative FBG load were not improved by the replacement or addition with other FBG indices.

Compared with FBG, indices representing long-term glycemic control, such as M-FBG and cumulative FBG load, were more closely related to DR risk and simultaneously had better discrimination. Previous studies have shown that chronic, long-term glycemic exposure is a critical risk factor for diabetic complications (25). Unlike FBG, which offers a snapshot, long-term glucose control indices consider time, highlighting the impact of chronically high glucose levels on DR development. Moreover, maintaining stable glucose levels over time plays a key role in management of DR. Our study showed that compared with M-FBG, cumulative FBG load was more strongly associated with the risk of DR. Furthermore, cumulative FBG load was superior to the M-FBG in improving AUC, IDI, NRI, AIC, and BIC when added to a model with classical risk factors. Studies have shown that the M-FBG level is a good predictor of the development/progression of DR (19). Moreover, patients with a high average glucose level have an increased likelihood of adverse associations (18, 26). However, the M-FBG considers only the FBG level and time. When the average FBG level is below the threshold, it does not lead to DR (28, 29). Compared to the M-FBG, the cumulative FBG load considers the intensity, time, and emphasizes the proportion of the FBG load (17). Simultaneously, the cumulative FBG load introduced a blood glucose reference standard for prediabetes and emphasized the impact of FBG levels above the threshold on the retina (24). Our findings are consistent with those of a previous study which showed that a fasting blood glucose level of above 5.6 mmol/L was associated with a higher risk of cardiovascular disease and all-cause mortality (30). Several studies on cumulative FBG load support our findings. Previous studies have shown that a higher cumulative FBG load is associated with a higher risk of DM complications (17, 31). From the perspective of a clinical utility, cumulative FBG load is a better predictor of DR risk, as minor alterations in risk predictions can have substantial effects when applied to large populations.

While our study demonstrated little improvement in discrimination for other models compared to cumulative FBG load, a separate study in type 2 diabetics found that the coexistence of high glycemic variability and high glucose levels may exacerbate the independent risk of premature mortality (32). This inconsistency with our results may be because glycemic variability mainly affects diabetic nephropathy (DN) rather than DR (33, 34). Although other FBG indices may have roles in some cases, our data suggests that a separate cumulative FBG load is adequate to predict the risk of DR. Therefore, as a simple measure of the level of FBG control at different time points, it can be considered for future risk prediction of DR.

Sensitivity analysis showed that the discrimination was similar among participants who had three or more FBG tests (n=5054 individuals), four or more FBG tests (n=3557 individuals), and five FBG tests (n=1587 individuals). The results showed that increasing the frequency of the FBG tests may not improve the prediction ability of these models. Therefore, from the perspective of the socioeconomic burden of the disease, appropriately reducing the frequency of FBG monitoring may not reduce the prediction efficiency.

This study is the first to use the cumulative FBG load to predict the risk of DR in a substantial community-based population. The strengths of this study include the use of detailed ophthalmic examinations, standardized questionnaires, biochemical analyses, and models that were fully adjusted for all common DR Risk factors. In addition, AUC was used to evaluate the model’s prediction performance, which IDI, NRI, AIC, BIC further complemented to alleviate the possible limitations of a single model evaluation indicator.

However, our study has some limitations. The correlation of FBG indices with DR severity remains unclear as we did not stage DR according to severity. Besides, this study did not offer the baseline levels and the progress of DR, and we cannot draw a causal association between FBG indices and the occurrence and progression of DR. Further exploration of FBG indices on DR occurrence and progression prediction may be the purpose of future research. Additionally, the study participants were all from the Jidong community, and the applicability of our results to other ethnic populations requires further investigation. In the case of continuous variables, there may be potential differences when the mean is used in place of missing data. Finally, the analysis did not include potential confounders such as creatinine, AL, diopters, and residual confounding factors.

## 6 Conclusions

In conclusion, our study supports the idea that a separate cumulative FBG load is perfectly adequate for capturing the glucose-related DR risk, and the predictive utility of cumulative FBG load is not further substantively improved by the addition or replacement with other FBG indices in the assessment of DR risk. Our findings highlight the importance of achieving long-term normal FBG levels in glycemic management.

## Data Availability

The raw data supporting the conclusions of this article will be made available by the authors, without undue reservation.
