# Supplementary material for: Risk prediction of diabetic retinopathy based on visit-to-visit fasting blood glucose indices
Source: Front Endocrinol (Lausanne). 2024 Sep 20;15:1420948. doi: 10.3389/fendo.2024.1420948 (PMC11449774; doi:10.3389/fendo.2024.1420948)

Supplementary Material

# Supplementary Tables

## Supplementary Table 1. Baseline characteristics across quartiles of FBG among 5054 participants.

|  | **Q1:**  **4.80(0.21)**  **n=1046** | **Q2:**  **5.26(0.11)**  **n=1354** | **Q3:**  **5.68(0.14)**  **n=1352** | **Q4:**  **7.41（2.18）n=1302** | ***P* value** | **P for Trend** |
| --- | --- | --- | --- | --- | --- | --- |
| Age, years | 43.06(10.79) | 44.56(10.78) | 45.70(10.64) | 51.17(11.88) | < 0.001 | < 0.001 |
| Female, n（%) | 648(61.95) | 777(57.39) | 642(47.49) | 553(42.47) | < 0.001 | < 0.001 |
| Educational level, n（%） |  |  |  |  | < 0.001 | < 0.001 |
| Illiteracy/Primary School/ Middle School | 241 (23.04) | 317 (23.41) | 366 (27.07) | 548 (42.09) |  |  |
| College/University | 805 (76.96) | 1037 (76.59) | 986 (72.93) | 754 (57.91) |  |  |
| Income, n（%） |  |  |  |  | < 0.001 | 0.33 |
| ≤5000 | 886 (84.70) | 1071 (79.10) | 1063 (78.62) | 1076 (82.64) |  |  |
| ＞5000 | 160 (15.30) | 283 (20.90) | 289 (21.38) | 226 (17.36) |  |  |
| Current smoking, n（%） | 152 (14.53) | 197 (14.55) | 262 (19.38) | 278 (21.35) | < 0.001 | < 0.001 |
| Current drinking, n（%） | 138 (13.19) | 237 (17.50) | 368 (27.22) | 353 (27.11) | < 0.001 | < 0.001 |
| Hypertension, n（%） | 146 (13.96) | 240 (17.73) | 347 (25.67) | 606 (46.54) | < 0.001 | < 0.001 |
| Dyslipidemia, n（%） | 471 (45.03) | 687 (50.74) | 798 (59.02) | 967 (74.27) | < 0.001 | < 0.001 |
| BMI, kg/m² | 23.44(3.21) | 23.99(3.32) | 24.81(3.33) | 25.82(3.50) | < 0.001 | < 0.001 |
| FBG, mmol/L | 4.80(0.21) | 5.26(0.11) | 5.68(0.14) | 7.41(2.18) | < 0.001 | < 0.001 |
| SD, mmol/L | 0.49(0.32) | 0.45(0.22) | 0.51(0.22) | 1.07(0.96) | < 0.001 | < 0.001 |
| CV, % | 9.59(4.72) | 8.51(3.63) | 9.18(3.62) | 14.71(8.88) | < 0.001 | < 0.001 |
| VIM, % | 0.82(0.34) | 0.69(0.28) | 0.71(0.28) | 0.82(0.35) | < 0.001 | 0.19 |
| ARV, % | 13.84(7.24) | 11.77(5.84) | 11.69(5.60) | 16.37(10.31) | < 0.001 | < 0.001 |
| M-FBG, mmol/L | 5.05(0.34) | 5.26(0.28) | 5.49(0.33) | 6.72(1.65) | < 0.001 | < 0.001 |
| cumulative FBG load, % | 1.49(3.08) | 1.82(2.88) | 3.22(3.69) | 15.92(14.01) | < 0.001 | < 0.001 |

Abbreviations: BMI, body mass index; FBG, fasting blood glucose; SD, standard deviation; CV, coefficient of variation; VIM, variation independent of the mean; ARV, average real variability; M-FBG, mean fasting blood glucose level; Q, quartile.

## Supplementary Table 2. Baseline characteristics across quartiles of SD among 5054 participants.

|  | **Q1:**  **0.27(0.08)**  **n=1263** | **Q2:**  **0.44(0.04)**  **n=1264** | **Q3:**  **0.59(0.05)**  **n=1264** | **Q4:**  **1.23(0.93)**  **n=1263** | ***P* value** | **P for Trend** |
| --- | --- | --- | --- | --- | --- | --- |
| Age, years | 44.77(10.39) | 45.36(10.96) | 45.75(11.40) | 49.14(12.43) | < 0.001 | < 0.001 |
| Female, n（%) | 728(57.64) | 691(54.67) | 667(52.77) | 534(42.28) | < 0.001 | < 0.001 |
| Educational level, n（%） |  |  |  |  | < 0.001 | < 0.001 |
| Illiteracy/Primary School/ Middle School | 302(23.91) | 326(25.79) | 359(28.40) | 485(38.40) |  |  |
| College/University | 961(76.09) | 938(74.21) | 905(71.60) | 778(61.60) |  |  |
| Income, n（%） |  |  |  |  | 0.09 | 0.01 |
| ≤5000 | 998(79.02) | 1022(80.85) | 1028(81.33) | 1048(82.98) |  |  |
| ＞5000 | 265(20.98) | 242(19.15) | 236(18.67) | 215(17.02) |  |  |
| Current smoking, n（%） | 186(14.73) | 204(16.14) | 198(15.66) | 301(23.83) | < 0.001 | < 0.001 |
| Current drinking, n（%） | 226(17.89) | 255(20.17) | 285(22.55) | 330(26.13) | < 0.001 | < 0.001 |
| Hypertension, n（%） | 243(19.24) | 295(23.34) | 314(24.84) | 487(38.56) | < 0.001 | < 0.001 |
| Dyslipidemia, n（%） | 653(51.70) | 684(54.11) | 736(58.23) | 850(67.30) | < 0.001 | < 0.001 |
| BMI, kg/m² | 23.99(3.35) | 24.28(3.32) | 24.58(3.42) | 25.41(3.59) | < 0.001 | < 0.001 |
| FBG, mmol/L | 5.34(0.46) | 5.41(0.51) | 5.58(0.63) | 7.01(2.46) | < 0.001 | < 0.001 |
| SD, mmol/L | 0.27(0.08) | 0.44(0.04) | 0.59(0.05) | 1.23(0.93) | < 0.001 | < 0.001 |
| CV, % | 5.13(1.5) | 8.35(0.92) | 10.89(1.15) | 17.68(7.97) | < 0.001 | < 0.001 |
| VIM, % | 0.42(0.14) | 0.68(0.12) | 0.86(0.17) | 1.06(0.34) | < 0.001 | < 0.001 |
| ARV, % | 6.67(2.56) | 10.98(2.87) | 14.44(3.67) | 21.36(9.71) | < 0.001 | < 0.001 |
| M-FBG, mmol/L | 5.28(0.43) | 5.32(0.39) | 5.45(0.47) | 6.57(1.76) | < 0.001 | < 0.001 |
| cumulative FBG load, % | 1.26(3.06) | 2.00(3.12) | 4.04(4.30) | 15.73(14.29) | < 0.001 | < 0.001 |

Abbreviations: BMI, body mass index; FBG, fasting blood glucose; SD, standard deviation; CV, coefficient of variation; VIM, variation independent of the mean; ARV, average real variability; M-FBG, mean fasting blood glucose level; Q, quartile.

## Supplementary Table 3. Baseline characteristics across quartiles of CV among 5054 participants.

|  | **Q1:**  **5.09(1.46)**  **n=1263** | **Q2:**  **8.29(0.73)**  **n=1264** | **Q3:**  **10.84(0.83)**  **n=1264** | **Q4:**  **17.83(7.85)**  **n=1263** | ***P* value** | **P for Trend** |
| --- | --- | --- | --- | --- | --- | --- |
| Age, years | 45.42(10.70) | 45.59(11.11) | 45.80(11.27) | 48.21(12.41) | < 0.001 | < 0.001 |
| Female, n（%) | 721(57.09) | 668(52.85) | 680(53.80) | 551(43.63) | < 0.001 | < 0.001 |
| Educational level, n（%） |  |  |  |  | < 0.001 | < 0.001 |
| Illiteracy/Primary School/ Middle School | 311(24.62) | 351(27.77) | 358(28.32) | 452(35.79) |  |  |
| College/University | 952(75.38) | 913(72.23) | 906(71.68) | 811(64.21) |  |  |
| Income, n（%） |  |  |  |  | 0.34 | 0.10 |
| ≤5000 | 1006(79.65) | 1018(80.54) | 1039(82.20) | 1033(81.79) |  |  |
| ＞5000 | 257(20.35) | 246(19.46) | 225(17.80) | 230(18.21) |  |  |
| Current smoking, n（%） | 184(14.57) | 220(17.41) | 193(15.27) | 292(23.12) | < 0.001 | < 0.001 |
| Current drinking, n（%） | 236(18.69) | 262(20.73) | 265(20.97) | 333(26.37) | < 0.001 | < 0.001 |
| Hypertension, n（%） | 276(21.85) | 295(23.34) | 313(24.76) | 455(36.03) | < 0.001 | < 0.001 |
| Dyslipidemia, n（%） | 664(52.57) | 705(55.78) | 734(58.07) | 820(64.92) | < 0.001 | < 0.001 |
| BMI, kg/m² | 24.11(3.34) | 24.35(3.38) | 24.55(3.34) | 25.26(3.68) | < 0.001 | < 0.001 |
| FBG, mmol/L | 5.41(0.55) | 5.50(0.73) | 5.60(0.73) | 6.82(2.47) | < 0.001 | < 0.001 |
| SD, mmol/L | 0.27(0.08) | 0.45(0.07) | 0.59(0.07) | 1.22(0.93) | < 0.001 | < 0.001 |
| CV, % | 5.09(1.46) | 8.29(0.73) | 10.84(0.83) | 17.83(7.85) | < 0.001 | < 0.001 |
| VIM, % | 0.41(0.13) | 0.66(0.10) | 0.85(0.13) | 1.11(0.31) | < 0.001 | < 0.001 |
| ARV, % | 6.59(2.47) | 10.86(2.69) | 14.34(3.50) | 21.65(9.53) | < 0.001 | < 0.001 |
| M-FBG, mmol/L | 5.36(0.51) | 5.40(0.64) | 5.46(0.55) | 6.40(1.75) | < 0.001 | < 0.001 |
| cumulative FBG load, % | 1.82(4.24) | 2.76(5.08) | 4.26(5.25) | 14.19(14.47) | < 0.001 | < 0.001 |

Abbreviations: BMI, body mass index; FBG, fasting blood glucose; SD, standard deviation; CV, coefficient of variation; VIM, variation independent of the mean; ARV, average real variability; M-FBG, mean fasting blood glucose level; Q, quartile.

## Supplementary Table 4. Baseline characteristics across quartiles of VIM among 5054 participants.

|  | **Q1:**  **0.40(0.11)**  **n=1263** | **Q2:**  **0.64(0.05)**  **n=1264** | **Q3:**  **0.83(0.06)**  **n=1264** | **Q4:**  **1.17(0.25)**  **n=1263** | ***P* value** | **P for Trend** |
| --- | --- | --- | --- | --- | --- | --- |
| Age, years | 46.95(11.20) | 46.81(11.46) | 45.9(11.48) | 45.38(11.58) | 0.001 | < 0.001 |
| Female, n（%) | 683(54.08) | 665(52.61) | 655(51.82) | 617(48.85) | 0.06 | 0.009 |
| Educational level, n（%） |  |  |  |  | 0.49 | 0.38 |
| Illiteracy/Primary School/ Middle School | 372(29.45) | 386(30.54) | 352(27.85) | 362(28.66) |  |  |
| College/University | 891(70.55) | 878(69.46) | 912(72.15) | 901(71.34) |  |  |
| Income, n（%） |  |  |  |  | 0.27 | 0.65 |
| ≤5000 | 1016(80.44) | 1035(81.88) | 1040(82.28) | 1005(79.57) |  |  |
| ＞5000 | 247(19.56) | 229(18.12) | 224(17.72) | 258(20.43) |  |  |
| Current smoking, n（%） | 207(16.39) | 215(17.01) | 193(15.27) | 274(21.69) | < 0.001 | 0.003 |
| Current drinking, n（%） | 253(20.03) | 265(20.97) | 283(22.39) | 295(23.36) | 0.18 | 0.03 |
| Hypertension, n（%） | 332(26.29) | 335(26.50) | 330(26.11) | 342(27.08) | 0.95 | 0.72 |
| Dyslipidemia, n（%） | 720(57.01) | 737(58.31) | 721(57.04) | 745(58.99) | 0.69 | 0.45 |
| BMI, kg/m² | 24.38(3.37) | 24.51(3.29) | 24.59(3.41) | 24.79(3.76) | 0.02 | 0.003 |
| FBG, mmol/L | 5.75(1.27) | 5.79(1.29) | 5.70(1.10) | 6.09(2.05) | < 0.001 | < 0.001 |
| SD, mmol/L | 0.33(0.31) | 0.53(0.42) | 0.65(0.43) | 1.02(0.84) | < 0.001 | < 0.001 |
| CV, % | 5.48(2.48) | 8.84(3.05) | 11.16(3.31) | 16.56(7.84) | < 0.001 | < 0.001 |
| VIM, % | 0.40(0.11) | 0.64(0.05) | 0.83(0.06) | 1.17(0.25) | < 0.001 | < 0.001 |
| ARV, % | 7.02(3.47) | 11.28(4.17) | 14.56(5.08) | 20.59(9.20) | < 0.001 | < 0.001 |
| M-FBG, mmol/L | 5.69(1.23) | 5.66(1.08) | 5.57(0.91) | 5.70(1.12) | 0.007 | 0.73 |
| cumulative FBG load, % | 4.51(10.01) | 5.13(9.35) | 5.23(8.27) | 8.15(10.71) | < 0.001 | < 0.001 |

Abbreviations: BMI, body mass index; FBG, fasting blood glucose; SD, standard deviation; CV, coefficient of variation; VIM, variation independent of the mean; ARV, average real variability; M-FBG, mean fasting blood glucose level; Q, quartile.

## Supplementary Table 5. Baseline characteristics across quartiles of ARV among 5054 participants.

|  | **Q1:**  **5.96(1.81)**  **n=1263** | **Q2:**  **10.21(1.06)**  **n=1264** | **Q3:**  **14.19(1.34)**  **n=1264** | **Q4:**  **23.09(8.48)**  **n=1263** | ***P* value** | **P for Trend** |
| --- | --- | --- | --- | --- | --- | --- |
| Age, years | 45.39(10.60) | 45.93(10.84) | 46.23(11.67) | 47.47(12.48) | < 0.001 | < 0.001 |
| Female, n（%) | 714(56.53) | 665(52.61) | 647(51.19) | 594(47.03) | < 0.001 | < 0.001 |
| Educational level, n（%） |  |  |  |  | < 0.001 | < 0.001 |
| Illiteracy/Primary School/ Middle School | 315(24.94) | 346(27.37) | 374(29.59) | 437(34.60) |  |  |
| College/University | 948(75.06) | 918(72.63) | 890(70.41) | 826(65.40) |  |  |
| Income, n（%） |  |  |  |  | 0.004 | 0.003 |
| ≤5000 | 1012(80.13) | 993(78.56) | 1029(81.41) | 1062(84.09) |  |  |
| ＞5000 | 251(19.87) | 271(21.44) | 235(18.59) | 201(15.91) |  |  |
| Current smoking, n（%） | 192(15.20) | 213(16.85) | 210(16.61) | 274(21.69) | 0.0001 | < 0.001 |
| Current drinking, n（%） | 238(18.84) | 274(21.68) | 277(21.91) | 307(24.31) | 0.01 | 0.001 |
| Hypertension, n（%） | 277(21.93) | 312(24.68) | 340(26.90) | 410(32.46) | < 0.001 | < 0.001 |
| Dyslipidemia, n（%） | 707(55.98) | 703(55.62) | 731(57.83) | 782(61.92) | 0.005 | 0.001 |
| BMI, kg/m² | 24.27(3.43) | 24.40(3.36) | 24.63(3.51) | 24.96(3.51) | 0.02 | < 0.001 |
| FBG, mmol/L | 5.50(0.60) | 5.68(0.89) | 5.70(1.03) | 6.45(2.46) | < 0.001 | < 0.001 |
| SD, mmol/L | 0.30(0.12) | 0.48(0.16) | 0.61(0.22) | 1.14(0.96) | < 0.001 | < 0.001 |
| CV, % | 5.60(2.09) | 8.73(2.22) | 10.85(2.64) | 16.87(8.31) | < 0.001 | < 0.001 |
| VIM, % | 0.45(0.17) | 0.68(0.17) | 0.83(0.18) | 1.07(0.31) | < 0.001 | < 0.001 |
| ARV, % | 5.96(1.81) | 10.21(1.06) | 14.19(1.34) | 23.09(8.48) | < 0.001 | < 0.001 |
| M-FBG, mmol/L | 5.38(0.52) | 5.48(0.73) | 5.54(0.82) | 6.22(1.69) | < 0.001 | < 0.001 |
| cumulative FBG load, % | 2.01(4.42) | 3.68(6.32) | 4.93(7.21) | 12.41(14.28) | < 0.001 | < 0.001 |

Abbreviations: BMI, body mass index; FBG, fasting blood glucose; SD, standard deviation; CV, coefficient of variation; VIM, variation independent of the mean; ARV, average real variability; M-FBG, mean fasting blood glucose level; Q, quartile.

## Supplementary Table 6. Baseline characteristics across quartiles of M-FBG among 5054 participants.

|  | **Q1:**  **4.93(0.16)**  **n=1258** | **Q2:**  **5.27(0.07)**  **n=1250** | **Q3:**  **5.54(0.10)**  **n=1283** | **Q4:**  **6.87(1.61)**  **n=1263** | ***P* value** | **P for Trend** |
| --- | --- | --- | --- | --- | --- | --- |
| Age, years | 42.06(9.57) | 44.32(10.54) | 46.02(10.98) | 52.59(11.80) | < 0.001 | < 0.001 |
| Female, n（%) | 795(63.20) | 682(54.56) | 616(48.01) | 527(41.73) | < 0.001 | < 0.001 |
| Educational level, n（%） |  |  |  |  | < 0.001 | < 0.001 |
| Illiteracy/Primary School/ Middle School | 233(18.52) | 297(23.76) | 366(28.53) | 576(45.61) |  |  |
| College/University | 1025(81.48) | 953(76.24) | 917(71.47) | 687(54.39) |  |  |
| Income, n（%） |  |  |  |  | < 0.001 | < 0.001 |
| ≤5000 | 978(77.74) | 991(79.28) | 1053(82.07) | 1074(85.04) |  |  |
| ＞5000 | 280(22.26) | 259(20.72) | 230(17.93) | 189(14.96) |  |  |
| Current smoking, n（%） | 177(14.07) | 203(16.24) | 224(17.46) | 285(22.57) | < 0.001 | < 0.001 |
| Current drinking, n（%） | 183(14.55) | 253(20.24) | 337(26.27) | 323(25.57) | < 0.001 | < 0.001 |
| Hypertension, n（%） | 160(12.72) | 250(20.00) | 337(26.27) | 592(46.87) | < 0.001 | < 0.001 |
| Dyslipidemia, n（%） | 558(44.36) | 673(53.84) | 757(59.00) | 935(74.03) | < 0.001 | < 0.001 |
| BMI, kg/m² | 23.48(3.24) | 24.21(3.54) | 24.90(3.33) | 25.67(3.36) | < 0.001 | < 0.001 |
| FBG, mmol/L | 5.02(0.36) | 5.35(0.36) | 5.65(0.41) | 7.31(2.29) | < 0.001 | < 0.001 |
| SD, mmol/L | 0.43(0.17) | 0.49(0.18) | 0.52(0.21) | 1.09(1.00) | < 0.001 | < 0.001 |
| CV, % | 8.77(3.49) | 9.24(3.45) | 9.38(3.75) | 14.66(9.58) | < 0.001 | < 0.001 |
| VIM, % | 0.79(0.31) | 0.75(0.28) | 0.71(0.28) | 0.78(0.37) | < 0.001 | 0.05 |
| ARV, % | 11.66(5.47) | 12.40(5.69) | 12.22(5.63) | 17.17(11.16) | < 0.001 | < 0.001 |
| M-FBG, mmol/L | 4.93(0.16) | 5.27(0.07) | 5.54(0.10) | 6.87(1.61) | < 0.001 | < 0.001 |
| cumulative FBG load, % | 0.31(0.81) | 1.48(1.59) | 3.68(2.11) | 17.52(13.44) | < 0.001 | < 0.001 |

Abbreviations: BMI, body mass index; FBG, fasting blood glucose; SD, standard deviation; CV, coefficient of variation; VIM, variation independent of the mean; ARV, average real variability; M-FBG, mean fasting blood glucose level; Q, quartile.

## Supplementary Table 7. Baseline characteristics across quartiles of cumulative FBG load among 5054 participants.

|  | **Q1:**  **0.00(0.00)**  **n=1285** | **Q2:**  **1.14(0.66)**  **n=1242** | **Q3:**  **4.03(1.14)**  **n=1264** | **Q4:**  **17.88(13.12)**  **n=1263** | ***P* value** | **P for Trend** |
| --- | --- | --- | --- | --- | --- | --- |
| Age, years | 42.37(9.37) | 45.05(10.74) | 45.87(11.20) | 51.79(12.19) | < 0.001 | < 0.001 |
| Female, n（%) | 807(62.80) | 672(54.11) | 615(48.66) | 526(41.65) | < 0.001 | < 0.001 |
| Educational level, n（%） |  |  |  |  | < 0.001 | < 0.001 |
| Illiteracy/Primary School/ Middle School | 232(18.05) | 326(26.25) | 355(28.09) | 559(44.26) |  |  |
| College/University | 1053(81.95) | 916(73.75) | 909(71.91) | 704(55.74) |  |  |
| Income, n（%） |  |  |  |  | < 0.001 | < 0.001 |
| ≤5000 | 988(76.89) | 996(80.19) | 1043(82.52) | 1069(84.64) |  |  |
| ＞5000 | 297(23.11) | 246(19.81) | 221(17.48) | 194(15.36) |  |  |
| Current smoking, n（%） | 180(14.01) | 204(16.43) | 221(17.48) | 284(22.49) | < 0.001 | < 0.001 |
| Current drinking, n（%） | 188(14.63) | 257(20.69) | 322(25.47) | 329(26.05) | < 0.001 | < 0.001 |
| Hypertension, n（%） | 179(13.93) | 261(21.01) | 326(25.79) | 573(45.37) | < 0.001 | < 0.001 |
| Dyslipidemia, n（%） | 594(46.23) | 683(54.99) | 735(58.15) | 911(72.13) | < 0.001 | < 0.001 |
| BMI, kg/m² | 23.50(3.29) | 24.33(3.37) | 24.82(3.42) | 25.62(3.43) | < 0.001 | < 0.001 |
| FBG, mmol/L | 5.10(0.34) | 5.38(0.39) | 5.60(0.50) | 7.26(2.33) | < 0.001 | < 0.001 |
| SD, mmol/L | 0.36(0.14) | 0.46(0.14) | 0.57(0.17) | 1.15(0.98) | < 0.001 | < 0.001 |
| CV, % | 7.17(2.91) | 8.73(2.88) | 10.41(3.32) | 15.76(9.12) | < 0.001 | < 0.001 |
| VIM, % | 0.64(0.28) | 0.72(0.26) | 0.80(0.28) | 0.87(0.38) | < 0.001 | < 0.001 |
| ARV, % | 9.17(4.29) | 11.64(4.72) | 13.90(5.51) | 18.78(10.80) | < 0.001 | < 0.001 |
| M-FBG, mmol/L | 4.99(0.21) | 5.29(0.18) | 5.51(0.20) | 6.84(1.63) | < 0.001 | < 0.001 |
| cumulative FBG load, % | 0.00(0.00) | 1.14(0.66) | 4.03(1.14) | 17.88(13.12) | < 0.001 | < 0.001 |

Abbreviations: BMI, body mass index; FBG, fasting blood glucose; SD, standard deviation; CV, coefficient of variation; VIM, variation independent of the mean; ARV, average real variability; M-FBG, mean fasting blood glucose level; Q, quartile.

## Supplementary Table 8. Spearman correlation coefficients r （and age- and sex-adjusted r） between each pair of FBG indices in participants (n=5054)

|  | **FBG** | **SD** | **CV** | **VIM** | **ARV** | **M-FBG** | **cumulative FBG load** |
| --- | --- | --- | --- | --- | --- | --- | --- |
| FBG | 1.000 |  |  |  |  |  |  |
| SD | 0.418(0.388) | 1.000 |  |  |  |  |  |
| CV | 0.301(0.282) | 0.979(0.981) | 1.000 |  |  |  |  |
| VIM | 0.009(0.022) | 0.809(0.829) | 0.896(0.907) | 1.000 |  |  |  |
| ARV | 0.096(0.074) | 0.829(0.831) | 0.856(0.855) | 0.793(0.799) | 1.000 |  |  |
| M-FBG | 0.786(0.758) | 0.455(0.427) | 0.305(0.286) | -0.048(-0.035) | 0.213(0.199) | 1.000 |  |
| cumulative FBG load | 0.677(0.638) | 0.702(0.693) | 0.580(0.580) | 0.264(0.295) | 0.488(0.491) | 0.895(0.881) | 1.000 |

Abbreviations: FBG, fasting blood glucose; SD, standard deviation; CV, coefficient of variation; VIM, variation independent of the mean; ARV, average real variability; M-FBG, mean fasting blood glucose level.

## Supplementary Table 9. Discrimination statistics for prediction of DR compared with the model with classical risk factors and FBG (n=5054)

| **Models** | **AUC (95%CI)** | **Changes in AUC（95%CI）** | **P value** |
| --- | --- | --- | --- |
| Classical risk factors + FBG | 0.8963(0.8721, 0.9205) | Reference |  |
| Classical risk factors + SD | 0.8717(0.8446, 0.8988) | -0.0246(-0.0403, -0.0089) | 0.002 |
| Classical risk factors + CV | 0.8494(0.8206, 0.8782) | -0.0469(-0.0667, -0.0272) | < 0.001 |
| Classical risk factors + VIM | 0.7721(0.7410, 0.8032) | -0.1242(-0.1504, -0.0980) | < 0.001 |
| Classical risk factors + ARV | 0.8249(0.7939, 0.8558) | -0.0714(-0.0940, -0.0489) | < 0.001 |
| Classical risk factors + M-FBG | 0.9105(0.8867, 0.9342) | 0.0142(0.0043, 0.0240) | 0.005 |
| Classical risk factors + cumulative FBG load | 0.9135(0.8890, 0.9380) | 0.0172(0.0042, 0.0302) | 0.009 |
| Classical risk factors + FBG + SD | 0.8993(0.8754, 0.9231) | 0.0030(-0.0022, 0.0082) | 0.26 |
| Classical risk factors + FBG + CV | 0.8958(0.8714, 0.9201) | -0.0005(-0.0012, 0.0002) | 0.13 |
| Classical risk factors + FBG + VIM | 0.8906(0.8641, 0.9171) | -0.0057(-0.0143, 0.0028) | 0.19 |
| Classical risk factors + FBG + ARV | 0.8976(0.8731, 0.9220) | 0.0013(-0.0037, 0.0062) | 0.61 |
| Classical risk factors + FBG + M-FBG | 0.9113(0.8877, 0.9348) | 0.0150(0.0059, 0.0240) | 0.001 |
| Classical risk factors + FBG + cumulative FBG load | 0.9139(0.8894, 0.9383) | 0.0176(0.0049, 0.0302) | 0.007 |
| Classical risk factors + SD + M-FBG | 0.9105(0.8868, 0.9342) | 0.0142(0.0044, 0.0240) | 0.004 |
| Classical risk factors + SD + cumulative FBG load | 0.9136(0.8889, 0.9383) | 0.0173(0.0039, 0.0307) | 0.01 |
| Classical risk factors + CV + M-FBG | 0.9106(0.8869, 0.9342) | 0.0143(0.0045, 0.0241) | 0.004 |
| Classical risk factors + CV + cumulative FBG load | 0.9140(0.8895, 0.9385) | 0.0177(0.0046, 0.0308) | 0.008 |
| Classical risk factors + VIM + M-FBG | 0.9105(0.8867, 0.9343) | 0.0142(0.0044, 0.0241) | 0.005 |
| Classical risk factors + VIM + cumulative FBG load | 0.9142(0.8899, 0.9385) | 0.0179(0.0050, 0.0309) | 0.007 |
| Classical risk factors + ARV + M-FBG | 0.9100(0.8861, 0.9339) | 0.0137(0.0038, 0.0237) | 0.007 |
| Classical risk factors + ARV + cumulative FBG load | 0.9148(0.8907, 0.9389) | 0.0185(0.0056, 0.0315) | 0.005 |
| Classical risk factors + FBG + SD + M-FBG | 0.9114(0.8878, 0.9351) | 0.0152(0.0061, 0.0242) | 0.001 |
| Classical risk factors + FBG + SD + cumulative FBG load | 0.9145(0.8899, 0.9390) | 0.0182(0.0052, 0.0311) | 0.006 |
| Classical risk factors + FBG + CV + M-FBG | 0.9112(0.8876, 0.9348) | 0.0149(0.0058, 0.024) | 0.001 |
| Classical risk factors + FBG + CV + cumulative FBG load | 0.9149(0.8906, 0.9391) | 0.0186(0.0059, 0.0312) | 0.004 |
| Classical risk factors + FBG + VIM + M-FBG | 0.9113(0.8877, 0.9348) | 0.0150(0.0058, 0.0241) | 0.001 |
| Classical risk factors + FBG + VIM + cumulative FBG load | 0.9150(0.8909, 0.9391) | 0.0187(0.0062, 0.0312) | 0.003 |
| Classical risk factors + FBG + ARV + M-FBG | 0.9109(0.8872, 0.9347) | 0.0146(0.0055, 0.0238) | 0.002 |
| Classical risk factors + FBG + ARV + cumulative FBG load | 0.9153(0.8914, 0.9393) | 0.0190(0.0065, 0.0316) | 0.003 |

Classical risk factors: age, sex, BMI, educational level, income, current smoking, current drinking, hypertension, dyslipidemia.

Abbreviations: DR, diabetic retinopathy; FBG, fasting blood glucose; AUC, area under the curve; CI, confidence interval; SD, standard deviation; CV, coefficient of variation; VIM, variation independent of the mean; ARV, average real variability; M-FBG, mean fasting blood glucose level.

## Supplementary Table 10. Reclassification statistics for prediction of DR compared with the model with classical risk factors and FBG (n=5054)

| **Models** | **IDI (95% CI)** | **P value** | **Categorical NRI (95%CI)** | **P value** |
| --- | --- | --- | --- | --- |
| Classical risk factors + FBG | Reference |  | Reference |  |
| Classical risk factors +SD | -0.0372(-0.0651, -0.0093) | 0.009 | -0.0751(-0.1465, -0.0037) | 0.04 |
| Classical risk factors +CV | -0.0861(-0.1173, -0.0549) | < 0.001 | -0.1615(-0.2292, -0.0938) | < 0.001 |
| Classical risk factors +VIM | -0.1618(-0.2016, -0.1221) | < 0.001 | -0.2442(-0.3120, -0.1763) | < 0.001 |
| Classical risk factors +ARV | -0.1030(-0.1413, -0.0647) | < 0.001 | -0.1788(-0.2530, -0.1047) | < 0.001 |
| Classical risk factors +M-FBG | 0.0501(0.0215, 0.0787) | < 0.001 | 0.1190(0.0418, 0.1963) | 0.003 |
| Classical risk factors +cumulative FBG load | 0.0603(0.0312, 0.0894) | < 0.001 | 0.1162(0.0295, 0.2028) | 0.009 |
| Classical risk factors + FBG + SD | -0.0003(-0.0053, 0.0048) | 0.92 | 0.0000(-0.0248, 0.0248) | 1.00 |
| Classical risk factors + FBG + CV | 0.0005(0.0000, 0.0010) | 0.05 | 0.0000(0.0000, 0.0000) | 1.00 |
| Classical risk factors + FBG + VIM | 0.0126(0.0057, 0.0195) | < 0.001 | 0.0568(0.0129, 0.1006) | 0.01 |
| Classical risk factors + FBG + ARV | 0.0010(-0.0052, 0.0073) | 0.75 | -0.0065(-0.0394, 0.0263) | 0.70 |
| Classical risk factors + FBG + M-FBG | 0.0511(0.0275, 0.0747) | < 0.001 | 0.0882(0.0150, 0.1614) | 0.02 |
| Classical risk factors + FBG + cumulative FBG load | 0.0614(0.0347, 0.0881) | < 0.001 | 0.1288(0.0482, 0.2095) | 0.002 |
| Classical risk factors + SD + M-FBG | 0.0498(0.0216, 0.0780) | < 0.001 | 0.1125(0.0360, 0.1890) | 0.004 |
| Classical risk factors + SD + cumulative FBG load | 0.0614(0.0299, 0.0928) | < 0.001 | 0.1411(0.0502, 0.2320) | 0.002 |
| Classical risk factors + CV + MEAN | 0.0489(0.0219, 0.0759) | < 0.001 | 0.0872(0.0119, 0.1624) | 0.02 |
| Classical risk factors + CV + cumulative FBG load | 0.0623(0.0315, 0.0931) | < 0.001 | 0.1407(0.0515, 0.2299) | 0.002 |
| Classical risk factors + VIM + M-FBG | 0.0490(0.0217, 0.0764) | < 0.001 | 0.0998(0.0250, 0.1747) | 0.009 |
| Classical risk factors + VIM + cumulative FBG load | 0.0624(0.0323, 0.0924) | < 0.001 | 0.1350(0.0464, 0.2235) | 0.003 |
| Classical risk factors + ARV + M-FBG | 0.0496(0.0214, 0.0778) | < 0.001 | 0.1066(0.0289, 0.1843) | 0.007 |
| Classical risk factors + ARV + cumulative FBG load | 0.0618(0.0321, 0.0915) | < 0.001 | 0.1280(0.0383, 0.2177) | 0.005 |
| Classical risk factors + FBG + SD + M-FBG | 0.0521(0.0282, 0.0759) | < 0.001 | 0.1007(0.0279, 0.1734) | 0.007 |
| Classical risk factors + FBG + SD + cumulative FBG load | 0.0645(0.0368, 0.0922) | < 0.001 | 0.1543(0.0674, 0.2413) | < 0.001 |
| Classical risk factors + FBG + CV + M-FBG | 0.0507(0.0272, 0.0742) | < 0.001 | 0.0878(0.0146, 0.1610) | 0.02 |
| Classical risk factors + FBG + CV + cumulative FBG load | 0.0652(0.0376, 0.0928) | < 0.001 | 0.1664(0.0800, 0.2527) | < 0.001 |
| Classical risk factors + FBG + VIM + M-FBG | 0.0506(0.0271, 0.0742) | < 0.001 | 0.0878(0.0146, 0.1610) | 0.02 |
| Classical risk factors + FBG + VIM + cumulative FBG load | 0.0643(0.0370, 0.0916) | < 0.001 | 0.1415(0.0576, 0.2254) | 0.001 |
| Classical risk factors + FBG + ARV + M-FBG | 0.0508(0.0272, 0.0744) | < 0.001 | 0.0880(0.0148, 0.1612) | 0.02 |
| Classical risk factors + FBG + ARV + cumulative FBG load | 0.0630(0.0358, 0.0901) | < 0.001 | 0.1278(0.0434, 0.2122) | 0.003 |

Classical risk factors: age, sex, BMI, educational level, income, current smoking, current drinking, hypertension, dyslipidemia.

Abbreviations: DR, diabetic retinopathy; FBG, fasting blood glucose; IDI, integrated discrimination improvement; CI, confidence interval; NRI, net reclassification improvement indexes; SD, standard deviation; CV, coefficient of variation; VIM, variation independent of the mean; ARV, average real variability; M-FBG, mean fasting blood glucose level.

## Supplementary Table 11. Improvement in goodness of model fit for prediction of DR compared with the model with classical risk factors and FBG (n=5054)

| **Models** | **AIC** | **BIC** | **ΔAIC** | **ΔBIC** |
| --- | --- | --- | --- | --- |
| Classical risk factors + FBG | 1037.0340 | 1108.8410 | Reference | Reference |
| Classical risk factors +SD | 1090.1180 | 1161.9250 | 53.0840 | 53.0840 |
| Classical risk factors +CV | 1149.6460 | 1221.4540 | 112.6120 | 112.6130 |
| Classical risk factors +VIM | 1285.9280 | 1357.7360 | 248.8940 | 248.8950 |
| Classical risk factors +ARV | 1185.1810 | 1256.9880 | 148.1470 | 148.1470 |
| Classical risk factors +M-FBG | 971.7281 | 1043.5350 | -65.3059 | -65.3060 |
| Classical risk factors +cumulative FBG load | 935.4598 | 1007.2670 | -101.5742 | -101.5740 |
| Classical risk factors + FBG + SD | 1036.4220 | 1114.7570 | -0.6120 | 5.9160 |
| Classical risk factors + FBG + CV | 1039.0050 | 1117.3400 | 1.9710 | 8.4990 |
| Classical risk factors + FBG + VIM | 1029.8340 | 1108.1690 | -7.2000 | -0.6720 |
| Classical risk factors + FBG + ARV | 1034.4700 | 1112.8050 | -2.5640 | 3.9640 |
| Classical risk factors + FBG + M-FBG | 970.5937 | 1048.9290 | -66.4403 | -59.9120 |
| Classical risk factors + FBG + cumulative FBG load | 936.8236 | 1015.1590 | -100.2104 | -93.6820 |
| Classical risk factors + SD + M-FBG | 973.6558 | 1051.9910 | -63.3782 | -56.8500 |
| Classical risk factors + SD + cumulative FBG load | 935.6610 | 1013.9960 | -101.3730 | -94.8450 |
| Classical risk factors + CV + MEAN | 972.5813 | 1050.9170 | -64.4527 | -57.9240 |
| Classical risk factors + CV + cumulative FBG load | 936.3731 | 1014.7080 | -100.6609 | -94.1330 |
| Classical risk factors + VIM + M-FBG | 972.7082 | 1051.0430 | -64.3258 | -57.7980 |
| Classical risk factors + VIM + cumulative FBG load | 936.7528 | 1015.0880 | -100.2812 | -93.7530 |
| Classical risk factors + ARV + M-FBG | 973.2932 | 1051.6280 | -63.7408 | -57.2130 |
| Classical risk factors + ARV + cumulative FBG load | 936.1218 | 1014.4570 | -100.9122 | -94.3840 |
| Classical risk factors + FBG + SD + M-FBG | 972.1164 | 1056.9800 | -64.9176 | -51.8610 |
| Classical risk factors + FBG + SD + cumulative FBG load | 935.1032 | 1019.9660 | -101.9308 | -88.8750 |
| Classical risk factors + FBG + CV + M-FBG | 972.5394 | 1057.4030 | -64.4946 | -51.4380 |
| Classical risk factors + FBG + CV + cumulative FBG load | 936.6577 | 1021.5210 | -100.3763 | -87.3200 |
| Classical risk factors + FBG + VIM + M-FBG | 972.4942 | 1057.3570 | -64.5398 | -51.4840 |
| Classical risk factors + FBG + VIM + cumulative FBG load | 937.7191 | 1022.5820 | -99.3149 | -86.2590 |
| Classical risk factors + FBG + ARV + M-FBG | 972.4456 | 1057.3090 | -64.5884 | -51.5320 |
| Classical risk factors + FBG + ARV + cumulative FBG load | 937.3807 | 1022.2440 | -99.6533 | -86.5970 |

Classical risk factors: age, sex, BMI, educational level, income, current smoking, current drinking, hypertension, dyslipidemia.

Abbreviations: DR, diabetic retinopathy; FBG, fasting blood glucose; AIC, Akaike Information Criterion; BIC, Bayesian Information Criterion; SD, standard deviation; CV, coefficient of variation; VIM, variation independent of the mean; ARV, average real variability; M-FBG, mean fasting blood glucose level.

## Supplementary Table 12. Discrimination statistics for prediction of DR compared with the model with classical risk factors and cumulative FBG load (n=5054)

| **Models** | **AUC (95%CI)** | **Changes in AUC（95%CI）** | **P value** |
| --- | --- | --- | --- |
| Classical risk factors + cumulative FBG load | 0.9135(0.8890, 0.9380) | Reference |  |
| Classical risk factors + cumulative FBG load + FBG | 0.9139(0.8894, 0.9383) | +0.0003(-0.0002, 0.0009) | 0.25 |
| Classical risk factors + cumulative FBG load + SD | 0.9136(0.8889, 0.9383) | +0.0001(-0.0010, 0.0012) | 0.85 |
| Classical risk factors + cumulative FBG load + CV | 0.9140(0.8895, 0.9385) | +0.0005(-0.0007, 0.0017) | 0.43 |
| Classical risk factors + cumulative FBG load + VIM | 0.9142(0.8899, 0.9385) | +0.0007(-0.0010, 0.0024) | 0.40 |
| Classical risk factors + cumulative FBG load + ARV | 0.9148(0.8907, 0.9389) | +0.0013(0.0001, 0.0025) | 0.03 |
| Classical risk factors + cumulative FBG load + FBG + SD | 0.9145(0.8899, 0.9390) | +0.0009(-0.0010, 0.0028) | 0.34 |
| Classical risk factors + cumulative FBG load + FBG + CV | 0.9149(0.8906, 0.9391) | +0.0013(-0.0006, 0.0033) | 0.19 |
| Classical risk factors + cumulative FBG load + FBG + VIM | 0.9150(0.8909, 0.9391) | +0.0015(-0.0009, 0.0038) | 0.22 |
| Classical risk factors + cumulative FBG load + FBG + ARV | 0.9153(0.8914, 0.9393) | +0.0018(0.0004, 0.0033) | 0.01 |

Classical risk factors: age, sex, BMI, educational level, income, current smoking, current drinking, hypertension, dyslipidemia.

Abbreviations: DR, diabetic retinopathy; FBG, fasting blood glucose; AUC, area under the curve; CI, confidence interval; SD, standard deviation; CV, coefficient of variation; VIM, variation independent of the mean; ARV, average real variability; M-FBG, mean fasting blood glucose level.

## Supplementary Table 13. Reclassification statistics for prediction of DR compared with the model with classical risk factors and cumulative FBG load (n=5054)

| **Models** | **IDI (95% CI)** | **P value** | **Categorical NRI (95% CI)** | **P value** |
| --- | --- | --- | --- | --- |
| Classical risk factors + cumulative FBG load | Reference |  | Reference |  |
| Classical risk factors + cumulative FBG load + FBG | 0.0011(-0.0018, 0.0040) | 0.46 | 0.0006(-0.0345, 0.0357) | 0.97 |
| Classical risk factors + cumulative FBG load + SD | 0.0011(-0.0041, 0.0063) | 0.68 | 0.0063(-0.0309, 0.0436) | 0.74 |
| Classical risk factors + cumulative FBG load + CV | 0.0020(-0.0016, 0.0055) | 0.28 | 0.0120(-0.0183, 0.0424) | 0.44 |
| Classical risk factors + cumulative FBG load + VIM | 0.0020(-0.0001, 0.0042) | 0.06 | 0.0125(-0.0123, 0.0372) | 0.32 |
| Classical risk factors + cumulative FBG load + ARV | 0.0015(-0.0024, 0.0054) | 0.45 | -0.0069(-0.0398, 0.0259) | 0.68 |
| Classical risk factors + cumulative FBG load + FBG + SD | 0.0042(-0.0038, 0.0122) | 0.30 | 0.0259(-0.0204, 0.0722) | 0.27 |
| Classical risk factors + cumulative FBG load + FBG + CV | 0.0049(-0.0009, 0.0107) | 0.10 | 0.0382(-0.0044, 0.0808) | 0.08 |
| Classical risk factors + cumulative FBG load + FBG + VIM | 0.0040(0.0001, 0.0080) | 0.05 | 0.0131(-0.0220, 0.0481) | 0.47 |
| Classical risk factors + cumulative FBG load + FBG + ARV | 0.0027(-0.0022, 0.0076) | 0.28 | -0.0006(-0.0399, 0.0386) | 0.98 |

Classical risk factors: age, sex, BMI, educational level, income, current smoking, current drinking, hypertension, dyslipidemia.

Abbreviations: DR, diabetic retinopathy; FBG, fasting blood glucose; IDI, integrated discrimination improvement; CI, confidence interval; NRI, net reclassification improvement indexes; SD, standard deviation; CV, coefficient of variation; VIM, variation independent of the mean; ARV, average real variability; M-FBG, mean fasting blood glucose level.

## Supplementary Table 14. Improvement in goodness of model fit for prediction of DR compared with the model with classical risk factors and cumulative FBG load (n=5054)

| **Models** | **AIC** | **BIC** | **ΔAIC** | **ΔBIC** |
| --- | --- | --- | --- | --- |
| Classical risk factors +cumulative FBG load | 935.4598 | 1007.2670 | Reference | Reference |
| Classical risk factors + FBG + cumulative FBG load | 936.8236 | 1015.1590 | 1.3638 | 7.8920 |
| Classical risk factors + SD + cumulative FBG load | 935.6610 | 1013.9960 | 0.2012 | 6.7290 |
| Classical risk factors + CV + cumulative FBG load | 936.3731 | 1014.7080 | 0.9133 | 7.4410 |
| Classical risk factors + VIM + cumulative FBG load | 936.7528 | 1015.0880 | 1.2930 | 7.8210 |
| Classical risk factors + ARV + cumulative FBG load | 936.1218 | 1014.4570 | 0.6620 | 7.1900 |
| Classical risk factors + FBG + SD + cumulative FBG load | 935.1032 | 1019.9660 | -0.3566 | 12.6990 |
| Classical risk factors + FBG + CV + cumulative FBG load | 936.6577 | 1021.5210 | 1.1979 | 14.2540 |
| Classical risk factors + FBG + VIM + cumulative FBG load | 937.7191 | 1022.5820 | 2.2593 | 15.3150 |
| Classical risk factors + FBG + ARV + cumulative FBG load | 937.3807 | 1022.2440 | 1.9209 | 14.9770 |

Classical risk factors: age, sex, BMI, educational level, income, current smoking, current drinking, hypertension, dyslipidemia.

Abbreviations: DR, diabetic retinopathy; FBG, fasting blood glucose; AIC, Akaike Information Criterion; BIC, Bayesian Information Criterion; SD, standard deviation; CV, coefficient of variation; VIM, variation independent of the mean; ARV, average real variability; M-FBG, mean fasting blood glucose level.

## Supplementary Table 15. Discrimination statistics for prediction of DR compared with the model with classical risk factors and FBG (n=3557)

| **Models** | **AUC (95%CI)** |
| --- | --- |
| Classical risk factors + FBG | 0.9058(0.8788, 0.9329) |
| Classical risk factors + SD | 0.8820(0.8511, 0.9129) |
| Classical risk factors + CV | 0.8587(0.8254, 0.8921) |
| Classical risk factors + VIM | 0.7733(0.7362, 0.8104) |
| Classical risk factors + ARV | 0.8336(0.7976, 0.8696) |
| Classical risk factors + M-FBG | 0.9167(0.8902, 0.9433) |
| Classical risk factors + cumulative FBG load | 0.9209(0.8937, 0.9480) |
| Classical risk factors + FBG + SD | 0.9073(0.8804, 0.9342) |
| Classical risk factors + FBG + CV | 0.9051(0.8779, 0.9323) |
| Classical risk factors + FBG + VIM | 0.9005(0.8714, 0.9297) |
| Classical risk factors + FBG + ARV | 0.9074(0.8797, 0.9350) |
| Classical risk factors + FBG + M-FBG | 0.9178(0.8915, 0.9441) |
| Classical risk factors + FBG + cumulative FBG load | 0.9213(0.8943, 0.9483) |
| Classical risk factors + SD + M-FBG | 0.9167(0.8902, 0.9433) |
| Classical risk factors + SD + cumulative FBG load | 0.9208(0.8933, 0.9484) |
| Classical risk factors + CV + M-FBG | 0.9171(0.8907, 0.9435) |
| Classical risk factors + CV + cumulative FBG load | 0.9211(0.8939, 0.9483) |
| Classical risk factors + VIM + M-FBG | 0.9171(0.8906, 0.9436) |
| Classical risk factors + VIM + cumulative FBG load | 0.9213(0.8943, 0.9483) |
| Classical risk factors + ARV + M-FBG | 0.9168(0.8901, 0.9434) |
| Classical risk factors + ARV + cumulative FBG load | 0.9216(0.8947, 0.9484) |
| Classical risk factors + FBG + SD + M-FBG | 0.9177(0.8912, 0.9443) |
| Classical risk factors + FBG + SD + cumulative FBG load | 0.9215(0.8940, 0.9490) |
| Classical risk factors + FBG + CV + M-FBG | 0.9176(0.8913, 0.9440) |
| Classical risk factors + FBG + CV + cumulative FBG load | 0.9220(0.8951, 0.9490) |
| Classical risk factors + FBG + VIM + M-FBG | 0.9178(0.8915, 0.9441) |
| Classical risk factors + FBG + VIM + cumulative FBG load | 0.9220(0.8952, 0.9487) |
| Classical risk factors + FBG + ARV + M-FBG | 0.9177(0.8913, 0.9442) |
| Classical risk factors + FBG + ARV + cumulative FBG load | 0.9219(0.8951, 0.9486) |

Classical risk factors: age, sex, BMI, educational level, income, current smoking, current drinking, hypertension, dyslipidemia.

Abbreviations: DR, diabetic retinopathy; FBG, fasting blood glucose; AUC, area under the curve; CI, confidence interval; SD, standard deviation; CV, coefficient of variation; VIM, variation independent of the mean; ARV, average real variability; M-FBG, mean fasting blood glucose level.

## Supplementary Table 16. Discrimination statistics for prediction of DR compared with the model with classical risk factors and FBG (n=1587)

| **Models** | **AUC (95%CI)** |
| --- | --- |
| Classical risk factors + FBG | 0.9024(0.8596, 0.9453) |
| Classical risk factors + SD | 0.8831(0.8417, 0.9245) |
| Classical risk factors + CV | 0.8650(0.8191, 0.9110) |
| Classical risk factors + VIM | 0.7695(0.7145, 0.8244) |
| Classical risk factors + ARV | 0.8207(0.7679, 0.8734) |
| Classical risk factors + M-FBG | 0.9210(0.8865, 0.9556) |
| Classical risk factors + cumulative FBG load | 0.9317(0.9004, 0.9631) |
| Classical risk factors + FBG + SD | 0.9039(0.8627, 0.9451) |
| Classical risk factors + FBG + CV | 0.9031(0.8607, 0.9455) |
| Classical risk factors + FBG + VIM | 0.8934(0.8443, 0.9426) |
| Classical risk factors + FBG + ARV | 0.9043(0.8636, 0.9450) |
| Classical risk factors + FBG + M-FBG | 0.9192(0.8828, 0.9556) |
| Classical risk factors + FBG + cumulative FBG load | 0.9308(0.8989, 0.9627) |
| Classical risk factors + SD + M-FBG | 0.9212(0.8868, 0.9555) |
| Classical risk factors + SD + cumulative FBG load | 0.9332(0.9017, 0.9647) |
| Classical risk factors + CV + M-FBG | 0.9228(0.8899, 0.9558) |
| Classical risk factors + CV + cumulative FBG load | 0.9321(0.9003, 0.9640) |
| Classical risk factors + VIM + M-FBG | 0.9241(0.8916, 0.9565) |
| Classical risk factors + VIM + cumulative FBG load | 0.9317(0.9000, 0.9634) |
| Classical risk factors + ARV + M-FBG | 0.9208(0.8860, 0.9555) |
| Classical risk factors + ARV + cumulative FBG load | 0.9323(0.9002, 0.9644) |
| Classical risk factors + FBG + SD + M-FBG | 0.9179(0.8794, 0.9565) |
| Classical risk factors + FBG + SD + cumulative FBG load | 0.9319(0.8991, 0.9647) |
| Classical risk factors + FBG + CV + M-FBG | 0.9188(0.8820, 0.9556) |
| Classical risk factors + FBG + CV + cumulative FBG load | 0.9307(0.8977, 0.9637) |
| Classical risk factors + FBG + VIM + M-FBG | 0.9202(0.8844, 0.9559) |
| Classical risk factors + FBG + VIM + cumulative FBG load | 0.9304(0.8979, 0.9629) |
| Classical risk factors + FBG + ARV + M-FBG | 0.9189(0.8818, 0.9560) |
| Classical risk factors + FBG + ARV + cumulative FBG load | 0.9315(0.8989, 0.9641) |

Classical risk factors: age, sex, BMI, educational level, income, current smoking, current drinking, hypertension, dyslipidemia.

Abbreviations: DR, diabetic retinopathy; FBG, fasting blood glucose; AUC, area under the curve; CI, confidence interval; SD, standard deviation; CV, coefficient of variation; VIM, variation independent of the mean; ARV, average real variability; M-FBG, mean fasting blood glucose level.

# Supplementary Figures

## Supplementary Figure 1.


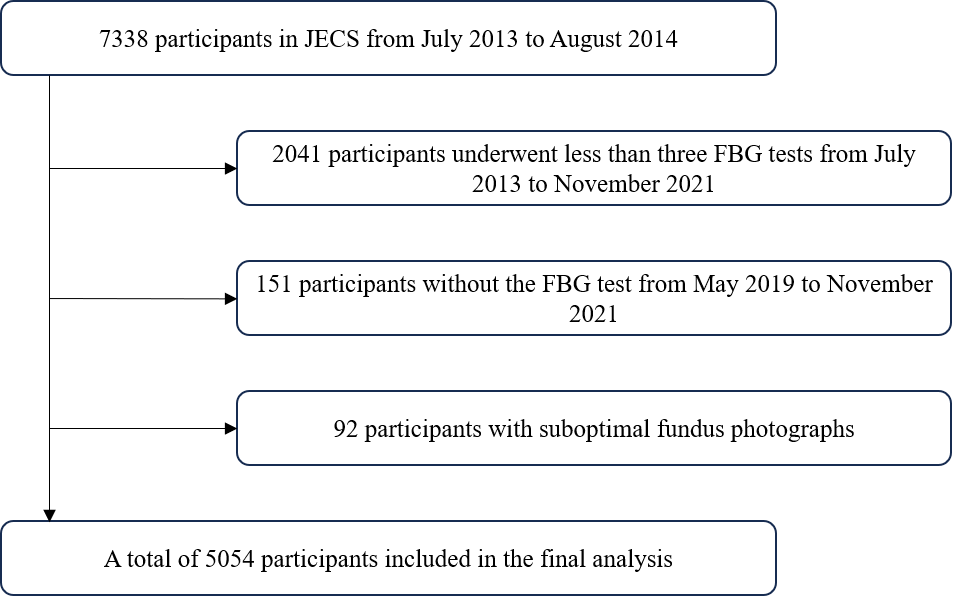

Supplement: Supplementary file 1 [file DataSheet1.docx]
